# Participants' preference for type of leaflet used to feed back the results of a randomised trial: a survey

**DOI:** 10.1186/1745-6215-11-116

**Published:** 2010-12-01

**Authors:** Stephen Brealey, Lazaros Andronis, Laura Dennis, Christine Atwell, Stirling Bryan, Simon Coulton, Helen Cox, Ben Cross, Fiona Fylan, Andrew Garratt, Fiona Gilbert, Maureen Gillan, Maggie Hendry, Kerenza Hood, Helen Houston, David King, Veronica Morton, Michael Robling, Ian Russell, Clare Wilkinson

**Affiliations:** 1 Department of Health Sciences, Seebohm Rowntree Building, University of York, Heslington, York YO10 5DD, UK; 2 Department of Health Economics, Occupational Health Building, University of Birmingham, Birmingham, West Midlands, B15 2TT, UK; 3 School of Health & Social Care, University of Teesside, Parkside West Offices, Middlesbrough, TS1 3BA, UK; 4 Department of Primary Care & Public Health, School of Medicine, Cardiff University, Neuadd Meirionydd, Heath Park, Cardiff, CF14 4YS, UK; 5 Department of Medicine, University of British Columbia, Centre for Clinical Epidemiology & Evaluation, 702 - 828 West 10th Avenue, Research Pavilion, Vancouver, BC V5Z 1M9, Canada; 6 Centre for Health Service Studies, Cornwallis George Allen Wing, University of Kent, Canterbury, Kent CT2 7NF, UK; 7 Department of Psychology, Leeds Metropolitan University, Leeds LS1 3HE, UK; 8 Norwegian Knowledge Centre for Health Services, PO Box 7004, St Olavs plass, N-0130 Oslo, Norway; 9 Department of Radiology, Lillian Sutton Building, University of Aberdeen, Foresterhill, Aberdeen, AB25 2ZD, UK; 10 Cardiff University School of Medicine, North Wales Clinical School, Gwenfro Building, Unit 5, Wrexham Technology Park, Wrexham, LL13 7YP, UK; 11 South East Wales Trials Unit, School of Medicine, Cardiff University, Neuadd Meirionydd, Heath Park, Cardiff, CF14 4YS, UK; 12 X-ray Department, York Hospital, Wigginton Road, York, YO31 8HE, UK; 13 School of Medicine, Swansea University, Swansea, SA2 8PP, UK

## Abstract

**Background:**

Hundreds of thousands of volunteers take part in medical research, but many will never hear from researchers about what the study revealed. There is a growing demand for the results of randomised trials to be fed back to research participants both for ethical research practice and for ensuring their co-operation in a trial. This study aims to determine participants' preferences for type of leaflet (short versus long) used to summarise the findings of a randomised trial; and to test whether certain characteristics explained participants' preferences.

**Methods:**

553 participants in a randomised trial about General Practitioners' access to Magnetic Resonance Imaging for patients presenting with suspected internal derangement of the knee were asked in the final follow-up questionnaire whether they would like to be fed back the results of the trial. Participants who agreed to this were included in a postal questionnaire survey asking about their preference, if any, between a short and a long leaflet and what it was about the leaflet that they preferred. Multinomial logistic regression was used to test whether certain demographics of responding participants along with treatment group explained whether a participant had a preference for type of leaflet or no preference.

**Results:**

Of the participants who returned the final follow-up questionnaire, 416 (88%) agreed to receive the results of the trial. Subsequently 132 (32%) participants responded to the survey. Most participants preferred the longer leaflet (55%) and the main reasons for this were the use of technical information (94%) and diagrams (89%). There was weak evidence to suggest that gender might explain whether participants have a preference for type of leaflet or not (P = 0.084).

**Conclusions:**

Trial participants want to receive feed back about the results and appear to prefer a longer leaflet. Males and females might require information to be communicated to them differently and should be the focus of further research.

**Trial registration:**

The trial is registered with http://www.isrctn.org/ and ID is ISRCTN76616358.

## Background

Every year hundreds of thousands of volunteers take part in medical research, but many will never hear from researchers about what the study revealed. There is now a growing demand for the results of randomised trials to be fed back to research participants [1,2]. Feeding back the results of trials has been described as an element of ethical research practice [3] and is a recommendation of the United Kingdom Department of Health's research governance framework [4]. This has also been described as a participant's right and that achieving their full co-operation in designing, conducting, reporting and implementing the results of research may ensure that they are the first people to hear the results [2]. Furthermore the majority of research participants want this right [5,6].

The results of trials, however, can be complicated, alarming and distressing [7]. The perceived negative psychological consequences of providing feedback may present a barrier to communicating study results [3,8]. Studies have shown that negative psychological reactions amongst participants include anger, anxiety, guilt or upset; although these studies also report participants being relieved or satisfied to receive trial results even if there is a potentially negative impact [7,9,10]. Despite the recent calls for research to be disseminated to participants and the potential for negative psychological impact there is currently little evidence advocating the most effective approach of communicating results - the preparation of such materials can be costly and time consuming [3,5].

In the DAMASK trial, we undertook a multi-centre pragmatic randomised trial to address the question of whether patients presenting to General Practitioners (GPs) with suspected internal derangement of the knee should be referred for early access to Magnetic Resonance Imaging (MRI) or directly to an orthopaedic specialist [11-13]. In view of the paucity of evidence to inform the method of communicating results to trial participants we thought it was timely to explore how participants might prefer to be fed back the results of a trial. The objectives of our survey were: to determine participants' preferences, if any, for type of leaflet (short versus long) which summarise the findings of the DAMASK trial; and to test whether certain demographics (e.g. gender, age) and treatment group explained whether the participants had a preference for type of leaflet or not.

## Methods

### Research Design

In the final follow-up questionnaire we asked the 553 participants in the DAMASK trial whether they would like to receive the results of the study. Participants who requested feedback of study results were then included in our survey after the results of the trial had been published in scientific journals [11-13]. This included being sent a cover letter asking the participants to take part in the survey, two leaflets (short and long) summarising the trial findings, and a one-page questionnaire. The short leaflet was a one-page summary in the style of an abstract that was written in plain language using bullet points, with minimum use of numbers, and no pictures or diagrams. The structure of this leaflet was presented as: background; aims of the study; how we did it; what we found; and conclusion. The longer leaflet was four pages in length which included a picture of MRI of the knee and two diagrams (a pie chart and bar chart) presenting results of the trial. The content of the leaflet was more technical with far greater use of numbers and percentages and explained the main results in terms of confidence intervals. The structure of this leaflet was presented as: background; aims of the study; how we did it; data collected; what we found; and should GP access to MRI of the knee be introduced into the NHS? Contact details of the trial co-ordinator were included at the end of each leaflet, copies of which are available on request from the corresponding author. Participants were asked to complete a one-page questionnaire about whether they preferred the one-page leaflet, the four-page leaflet or had no preference. If a participant preferred a type of leaflet they were asked (yes/no) whether it was due to: its length; the amount of technical information; the use of numbers; the use of percentages; the use of diagrams. A free text box was provided for participants to record other reasons about their preference for a leaflet.

### Analysis

To test for differences in baseline characteristics of participants who did and did not respond to completing the survey we used χ^2 ^tests and t-tests. Participants' preference for a type of leaflet or no preference and the reason for their preference are presented descriptively. Additional free text responses as to why participants preferred a type of leaflet were coded retrospectively. Multinomial logistic regression was used to test whether certain demographics of responding participants (i.e. gender [0 = female; 1 = male]; age [years], employment [0 = employed; 1 = other], age left education [years]) along with treatment group [0 = MRI (and Orthopaedic Referral); 1 = Orthopaedic Referral] explained their preference for a type of leaflet or not. This analysis was undertaken using Stata version 10.0 (Statacorp, Texas) [14] and included the commands *mlogit *and *mlogtest *[15].

### Research ethics

The DAMASK trial was designed to comply with the Declaration of Helsinki as adopted by the World Medical Association. The Northern and Yorkshire Multi-Centre Research Ethics Committee approved the trial (reference number MREC/1/3/59). Ethics approval was not necessary for this survey as we were advised that this was an audit of feeding back trial results to participants.

## Results

Of the 553 participants in the DAMASK trial, 471 (85%) completed the final follow-up questionnaire. For those participants who returned the final follow-up questionnaire, 416 (88%) agreed that they would like to receive the results of the study. Subsequently 132 (32%) participants responded to the survey about their preference for type of leaflet feeding back the results of the trial. Of these 132 participants, 73 (55%) were in the MRI (and orthopaedic referral) group and 59 (45%) were in the orthopaedic referral (control group).

Table 1 presents the baseline characteristics of participants who did and did not complete the questionnaire. Responders and non-responders were similar in key characteristics except for age (P < 0.001) and the emotional functioning sub-scale of the KQoL-26 questionnaire (P = 0.019).

**Table 1:** Participants' characteristics at baseline

Characteristics	Responders (n = 132)	Non-responders (n = 284)	P-Values
Sex: male, n (%)	78/132 (59.1)	177/284 (62.3)	0.529

Ethnicity: white, n (%)	127/129 (98.4)	274/277 (98.9)	0.691

Employed: yes, n (%)	112/126 (88.9)	240/261 (92.0)	0.325

Age, years			
n	132	284	<0.001
Mean (SD)	43.0 (9.4)	39.2 (10.3)	

KQoL-26 (0-100, 100 = best health):			
Physical functioning			
N	131	283	0.962
Mean (SD)	59.3 (19.4)	59.2 (19.2)	
Activity limitations			
N	130	283	0.374
Mean (SD)	52.9 (25.1)	50.5 (25.2)	
Emotional functioning			
N	131	284	0.019
Mean (SD)	45.7(22.1)	40.4 (21.1)	

SF-36 (mean = 50, SD = 10, 100 = best health):^a^			
Physical component			
N	129	267	0.088
Mean (SD)	40.0 (8.3)	38.4 (8.8)	
Mental component			
N	129	267	0.388
Mean (SD)	49.1 (10.9)	48.0 (11.4)	

EQ-5D (0-1, 1 = best health)			
N	131	283	0.064
Mean (SD)	0.62 (0.23)	0.57 (0.27)	

^a ^The SF-36 physical and mental health summary scales use norm-based scoring; 50 (SD = 10) is the general United States population mean

Table 2 presents participants' preferences for the one-page leaflet, four-page leaflet or no preference for a leaflet. Most participants preferred the four-page leaflet (55%). The main reasons for preferring this leaflet was use of technical information (94%) and diagrams (89%). Of the 73 participants who preferred the four-page leaflet, 31 (44%) recorded reasons in free text as to why they preferred this leaflet. This re-iterated the use of diagrams as a good visual aid and the provision of more detailed information which improved understanding. Only 21% of participants preferred the one-page who all recorded this was because of the length of the leaflet (100%); and 15 (54%) of them also recorded that their reason for choosing this leaflet was its shortness and simplicity although some suggested that it would benefit from the use of diagrams.

**Table 2:** Participants' preference for type of leaflet or no preference

Reason for preference	No preference n (%)	One-page leaflet n (%)	Four-page leaflet n (%)	Total n
Overall preference	31/132 (24)	28/132 (21)	73/132 (55)	132

a) Length	1/8 (12)	28/28 (100)	26/65 (40)	101

b) Technical information	5/8 (62)	9/25 (36)	64/68 (94)	101

c) Use of numbers	3/8 (37)	8/26 (31)	37/64 (58)	98

d) Use of percentages	4/8 (50)	6/26 (23)	47/66 (71)	100

e) Use of diagrams	5/8 (62)	2/26 (8)	63/71 (89)	105

Table 3 presents the results of the regression analysis that explored the effect of participant characteristics and treatment group on their preference for type of leaflet or no preference. The pseudo R^2 ^shows that only 4% of variation was explained by this model, though with adequate goodness-of-fit. None of the variables significantly explained participants' preferences in the comparisons between 'no preference for a leaflet and the four-page leaflet' and the 'one-page leaflet and the four-page leaflet'. The final comparison between the 'one-page leaflet and having no preference for a leaflet' did find that males compared to females were significantly less likely to prefer the one-page leaflet, Odds ratio (OR) = 0.263 (95% CI 0.079 to 0.880), P = 0.030.

**Table 3:** Multinomial logistic regression model for variables that explain participants' preference

Participant preference	Coefficient, B	Standard Error	P-Value	Exp(B)	95% CI for Exp(B)
*No preference Vs Four-page*					
Gender	0.656	0.518	0.205	1.927	0.698 to 5.317
Age	0.013	0.027	0.620	1.013	0.962 to 1.068
Education	-0.111	0.086	0.199	0.895	0.756 to 1.060
Employed	0.090	0.700	0.897	1.094	0.277 to 4.320
Treatment	0.080	0.460	0.862	1.083	0.440 to 2.666
Constant	0.013	2.101	0.995	1.013	0.016 to 62.231

*One-page Vs Four-page*					
Gender	-0.679	0.492	0.168	0.507	0.193 to 1.330
Age	0.027	0.027	0.317	1.027	0.974 to 1.084
Education	0.015	0.069	0.828	1.015	0.887 to 1.162
Employed	-0.686	0.866	0.428	0.504	0.092 to 2.748
Treatment	-0.396	0.490	0.419	0.673	0.257 to 1.759
Constant	-1.833	1.853	0.323	0.160	0.004 to 6.040

*One-page vs No preference*					
Gender	-1.335	0.616	0.030	0.263	0.079 to 0.880
Age	0.014	0.033	0.674	1.014	0.950 to 1.083
Education	0.126	0.098	0.199	1.134	0.516 to 1.373
Employed	-0.776	0.977	0.427	0.460	0.068 to 3.126
Treatment	-0.476	0.579	0.411	0.621	0.200 to 1.933
Constant	-1.846	2.476	0.456	0.158	0.001 to 20.230

Log likelihood = -115.5; number of observations = 121; likelihood ratio χ^2^-test statistic, 9.9 df = 10 (P = 0.445); pseudo R^2 ^= 0.041

Table 4 shows the results of the likelihood tests of the contribution of each independent variable to the regression model with only gender approaching a significant effect on preference for type of leaflet or no preference (χ^2 ^= 4.96, df = 2, P = 0.084).

**Table 4:** Likelihood ratio tests for independent variables

Participant preference	Chi-square	df	P-value
Gender	4.961	2	0.084
Age	1.101	2	0.577
Education	2.283	2	0.319
Employed	0.816	2	0.665
Treatment	0.823	2	0.663

## Discussion

Most participants (88%) wanted feed back of the DAMASK trial results which is comparable with findings from a literature review that a median of 90% (range 20%-100%) of participants wished to receive study results [5]. This range of patients wanting feedback is, however, very wide. Interestingly the review showed that for several studies of patients being treated for cancer, 90% of participants or more wanted to be informed of the study results [16-19]. In contrast, for studies of pregnant women only 20% [10] and 40% [20] of woman wanted study results. In our trial we asked participants about being fed back the study results at the final postal questionnaire follow-up, but it might be more appropriate to do this when enrolling patients to take part and include it in the patient information leaflet and consent of patients.

The majority of participants (55%) preferred the longer leaflet and the main reasons for this were the technical information provided and use of diagrams to explain trial results. In contrast, every participant who preferred the one-page leaflet gave length of the leaflet as the reason for this. In the free text responses it was clarified that the leaflet being short and simple was the reason for preferring the one-page leaflet although some suggested the leaflet would have benefited from the use of diagrams. Graphical formats are increasingly being used to present risk information to patients [21,22] and has been shown to improve their understanding of quantitative information over textual or written formats [23,24]. Our findings support the use of a longer leaflet with visual aids to explain trial results which in particular might benefit participants with reading difficulties or low numeracy skills. We did, however, only compare participants' preference for a one-page leaflet compared with a four-page leaflet. It is possible that a shorter leaflet such as two-pages with the use of diagrams would have been preferred.

The findings from the regression analyses suggest that gender can significantly influence preference for type of leaflet (one-page) or no preference (P = 0.030); although this was the only significant finding from the several explanatory analyses that were performed and thus possibly a false-positive result that should be interpreted with caution. Whilst taking this into consideration: 71% of participants who had no preference for a leaflet were male (29% female); 50% and 58% of males respectively preferred a one-page or four-page leaflet. These findings indicate that females were more interested in how the information about trial results was presented to them which is consistent with women using health services more than men and seeking more health information [25]. Therefore it might be appropriate in research that has a female population such as treatments for breast cancer or during pregnancy to consider feeding back results differently than for research including males such as the treatment for prostate cancer. Research about communicating results to participants of a cardiac rehabilitation trial has shown that gender can be important with women (97%) compared to men (76%) significantly more likely to prefer receiving study results by letter (P = 0.009); and no women (0%) compared to men (15%) preferring communication by email or the web (P = 0.024) [26]. The use of qualitative methods could help to further explore the communication of trial results to males and females. None of the other characteristics of the participants appeared to be important for influencing their preference for a type of leaflet or not. Including ethnicity as a variable in the analyses would have been desirable, but in our survey sample there were only two participants who were non-white (who both preferred the four-page leaflet). Therefore, it was not appropriate to include ethnicity in the model.

A limitation of our survey was the low response rate, although participants' characteristics were mostly similar between those who did and did not respond. Younger patients, however, were significantly less likely to respond, though there was only a small difference in years and, from the analyses, age was not important for explaining participants' preferences. Participants who scored lower on the emotional functioning sub-scale of the KQoL-26 questionnaire were also significantly less likely to respond. This might possibly be a chance finding although the emotional functioning sub-scale is concerned with participants feeling downhearted and low, angry and annoyed, or worried about their knee worsening. The trial findings indicated to participants that there were only small benefits from GP access to MRI in terms of the physical functioning of their knee. It is conceivable that participants who were downhearted and low or worried about their knee were dissatisfied with the limited benefits of the intervention and therefore were less willing to complete the survey. Alternatively, they could have been dissatisfied with the methods offered for feeding back the trial results. Research into communicating clinical trial results to participants in a study about Huntington's disease found that participants reported high or complete satisfaction with an investigator of the trial contacting individual participants by telephone to explain the results (89%) or participants' attendance to a conference call with study investigators (82%) but relatively low satisfaction with the sponsor's press release (50%) [27]. It is possible that the participants did not respond to our survey because they valued more personalised approaches to communicating the trial results. There was also a considerable delay from December 2006 which was when the last participant was followed-up in the trial to May 2009 when we informed the participants of the results of the trial. This was because we wanted to communicate the most accurate results possible to participants that had been peer-reviewed and accepted for publication in scientific journals. This delay might have contributed to participants' lack of interest in the survey.

Despite several studies commenting on the negative psychological impact of feeding back results of trials to participants [7,9,10], the trial co-ordinator has not had any contact with participants about the trial results. Nor did participants in the survey express any anxieties about the information conveyed. This may partly be due to the limited generalisability of this study, in that it is a single randomised trial including participants with a musculoskeletal condition which was not particularly acute or life-threatening and for which there was minimal difference in patient outcomes between the two groups. In a trial with a more vulnerable population (e.g. cancer patients) or with serious adverse events, participants could potentially have very different experiences which could affect their attitude towards the method of feeding back trial results. They might prefer a short leaflet that is plain and simple with clear messages such as "what these results mean to you" or "further steps you should take". It might also be important to present the findings separately for those who had a positive result compared with those who have had negative results, with the latter requiring a more personalised delivery of results through a telephone call or visit to the clinic [18]. Indeed, it might be appropriate to guide the participant back to their main care provider who can offer the necessary clinical advice in the context of the trial results. Our study also used a randomised trial design and was evaluating the effectiveness of a diagnostic test. Participants that have a different experience of research which use alternative study designs (e.g. surveys, cohorts) or an evaluation of different treatments might like the results of the study to be communicated to them differently. This study might also have benefited from the involvement of trial participants in the design and piloting of the leaflets which has been recommended for future research [28]. In addition to postal methods of feeding back results, other communication methods have been suggested including the telephone or a face-to-face consultation [6]. Using the postal method is likely to be less time consuming and costly than the additional salary required for a suitably trained and knowledgeable person to spend time explaining trial results to individual participants. In our study, it only cost £105 and £252 to print the one-page and four-page leaflets respectively and the cost of posting these materials was estimated to be £150. The cost per trial participant for sending out both leaflets was therefore £1.22. Finally randomised trials are increasingly making use of the internet and email to recruit participants, to maintain contact during the trial, to deliver the intervention and to collect data [29,30]. Participants could be asked when enrolled into a trial whether an electronic method could be used to feed back the results.

## Conclusions

Serious consideration should be given about how and to whom the results of the trial should be fed back to participants as part of good research practice [2-4]. This is important for sharing the results of the research to the patients for which they contributed and might improve their future care [8]. Participants in our trial did want to receive feed back about the trial results and appear to prefer the longer leaflet supported with graphs to present study findings and more technical information. It is possible that there could be a difference between males and females as to how information should be communicated to them and requires further research. Future research should also assess the effect of participants' preferences in different patient populations with more significant differences in outcomes and the effect of other modes of communicating trial findings on their understanding of results. Finally feeding back the results of trials to participants has the advantage of not only underpinning good research practice, but also appeals to participants' altruistic motives [31,32] and sharing study results might enhance their understanding of randomised trials [33]. This could have the advantage of improving recruitment and co-operation with data collection and ultimately the evidence-base to improve patient care.

## Competing interests

The authors declare that they have no competing interests.

## Authors' contributions

LD and SDB conceived the study and discussed its design with VM. LD collected the data. SDB analysed the data and drafted the manuscript. All authors read, commented on, and approved the final manuscript.
